# On systems and control approaches to therapeutic gain

**DOI:** 10.1186/1471-2407-6-104

**Published:** 2006-04-25

**Authors:** Tomas Radivoyevitch, Kenneth A Loparo, Robert C Jackson, W David Sedwick

**Affiliations:** 1 Department of Epidemiology and Biostatistics Case Western Reserve University, Cleveland, Ohio 44106, USA; 2 Department of Electrical Engineering and Computer Science Case Western Reserve University, Cleveland, Ohio 44106, USA; 3 Cyclacel Ltd., Dundee Technopole James Lindsay Place, Dundee, DD1 5JJ, UK; 4 Department of Hematology and Oncology Case Western Reserve University, Cleveland, Ohio 44106, USA

## Abstract

**Background:**

Mathematical models of cancer relevant processes are being developed at an increasing rate. Conceptual frameworks are needed to support new treatment designs based on such models.

**Methods:**

A modern control perspective is used to formulate two therapeutic gain strategies.

**Results:**

Two conceptually distinct therapeutic gain strategies are provided. The first is *direct *in that its goal is to kill cancer cells more so than normal cells, the second is *indirect *in that its goal is to achieve implicit therapeutic gains by transferring states of cancer cells of non-curable cases to a target state defined by the cancer cells of curable cases. The direct strategy requires models that connect anti-cancer agents to an endpoint that is modulated by the cause of the cancer and that correlates with cell death. It is an abstraction of a strategy for treating mismatch repair (MMR) deficient cancers with iodinated uridine (IUdR); IU-DNA correlates with radiation induced cell killing and MMR modulates the relationship between IUdR and IU-DNA because loss of MMR decreases the removal of IU from the DNA. The second strategy is indirect. It assumes that non-curable patient outcomes will improve if the states of their malignant cells are first transferred toward a state that is similar to that of a curable patient. This strategy is difficult to employ because it requires a model that relates drugs to determinants of differences in patient survival times. It is an abstraction of a strategy for treating BCR-ABL pro-B cell childhood leukemia patients using curable cases as the guides.

**Conclusion:**

Cancer therapeutic gain problem formulations define the purpose, and thus the scope, of cancer process modeling. Their abstractions facilitate considerations of alternative treatment strategies and support syntheses of learning experiences across different cancers.

## Background

Recent scientific research trends toward systems biology have brought new life to theoretical cancer research [1]. Mathematical models of cancer relevant processes, rare in the past [2,3], have now become common [4-7], and some appear to have predictive capabilities [8,9]. The ultimate goal is to use such models to design new cancer treatments.

In this paper we formulate two systems and control oriented abstractions of two conceptually distinct instances of therapeutic gain strategies, a direct one for mismatch repair deficient cancers, and an indirect one for BCR-ABL leukemias. The abstractions facilitate considerations of alternative treatment strategies and form the basis of a conceptual framework that supports syntheses of learning experiences across different cancers. The systems and control perspective that underlies these abstractions is reviewed briefly in this section.

### Simulation

Systems of first order nonlinear ordinary differential equations (ODEs) [10] are commonly used to model and simulate biochemical networks. The models have boundary condition nodes that decouple them from their environment, and state variable nodes that are responsive to environmental changes. The models are typically built and validated using 4 types of data:

1. *Configuration data *– These data specify how various system components are interconnected. Wiring diagrams are an excellent example of this form of data.

2. *Isolated component data *– These data describe how system components behave in complete isolation, e.g. enzyme mechanisms and estimates of binding constants.

3. *Operating point data *– These data describe the state of the interconnected system in steady state, e.g. metabolite concentrations in whole cell extracts of unperturbed cells.

4. *Dynamic response data *– These data specify how the interconnected system reacts to perturbations, e.g. metabolite concentration time course responses to drugs.

In general, configuration data, isolated component data and operating point data are used to build models, and dynamic response data are then used to validate them, e.g. see [3]. Once built, the models can be represented using Systems Biology Markup Language (SBML) [11] and analyzed using various software packages [12], such as R [13] and Matlab [14].

### Control

**Figure 1:**
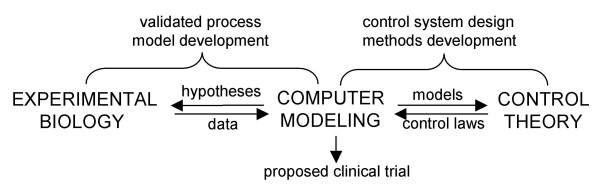
Treatment design via two interacting cycles, one in which models are developed iteratively through predictions followed by experimental validations, and a second in which control systems are developed iteratively through comparisons of new design performances in controller-model combined simulations. As these cycles evolve, the current best control law will be the current best proposal for a state feedback based clinical trial. The trials will be individualized via polymorphism-based model parameter perturbations and differences in initial states.

The inverse of simulation is control. The goal in control is to identify system inputs (boundary condition time courses in this paper) that will drive the system toward an objective, such as a target steady-state (a regulation problem) or a desired time course trajectory (a tracking problem). Various types of control methods can potentially be applied to problems of interest in cancer research, e.g. classical techniques such as proportional, integral and derivative (PID) control, or model-based methods which involve state estimation as a component of the feedback system. In model-based methods, the first step is to develop a validated process model, and the second step is to use the model and the overall problem objective to design a control strategy. In the future, as cancer treatment protocols are eventually developed based on models and control system design methodologies (Fig. 1), and as model parameters and initial states become individualized through patient specific laboratory measurements, treatment dose time course schedules will become individualized as a direct consequence of using model-based control system designs. Further, since successful control systems in nature and in industry are generally implemented using closed-loop feedback strategies, where current state estimates derived from available observations are used to determine the most suitable input values in the next time interval, as science advances and as measurement costs decrease, it is extremely likely that state feedback based cancer treatments will eventually become the standard of care. This suggests that systems cancer biology should perhaps focus first where it is most likely to perform the best, namely, on problems where control system techniques can most readily be applied. In this regard, leukemias are attractive to study because leukemic cells can be sampled to measure relevant molecular activities [15-22] in real time (i.e. daily), as needed to implement feedback control strategies. Leukemias are also attractive to study because they seem to be simpler than solid tumors, perhaps because white blood cells naturally leave and enter tissues and thus require fewer steps to become malignant, a notion supported by shorter latency times for radiation induced leukemias [23-25] versus solid tumors [26]. Since increased process understanding favors process controllability, leukemia simplicity relative to solid tumors should favor its curability.

In the past, control system design *methods *have been developed primarily for linear [27] rather than nonlinear systems [28], and most often for systems with rich rather than sparse time course data. New design methods and/or *ad hoc *solutions will therefore be needed to successfully apply control system approaches to cancer therapy.

Previous attempts to apply control theory [29,30] to cancer therapy [31,32] lacked sufficient data, and the process models that were used were not molecularly based. As our understanding of cancer relevant biological processes increases, our knowledge of the dynamic behavior of these processes will improve, and thus so too will the likelihood of successfully applying control system design approaches to cancer therapy. The purpose of this paper is to formulate (but not solve) two systems and control oriented, biochemical system model-based therapeutic gain strategies as abstractions of two conceptually distinct, specific cancer treatment strategies.

## Methods

SBMLR [33-35] was used with a folate model [3] to map the childhood leukemia diagnostic bone marrow microarray data of Yeoh et al [36] into predicted steady state fluxes of *de novo *purine synthesis (DNPS) and *de novo *thymidylate synthesis (DNTS), see [37]. Briefly, genes with multiple probe sets were represented by the set with the highest average value. These were then normalized within genes by dividing by the mean of the leukemia subtype medians. The normalization constants were then equated to the steady state of the folate model, and proportionality between mRNA and protein levels was then assumed to compute individualized steady states, i.e. as described in [37]. For additional details, R scripts used to produce Figs. 4 and 5 are available as supplementary material [38].

## Results

### Direct approach to therapeutic gain

**Figure 2:**
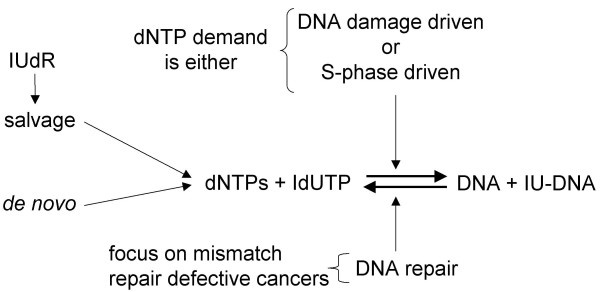
IUdR treatment of MMR defective cancers.

Iododeoxyuridine (IUdR) sensitization of cells to radiation induced cell killing [39,40] is proportional to its incorporation into DNA [41,42], and IU-DNA has a longer half-life in mismatch repair deficient (MMR^-^) cells than in MMR^+ ^cells [43,44]. Thus, IU-DNA levels at the time of irradiation correlate with the probability of clonogenic cell death and are modulated by the cause of the cancer. Suppose we have a model of IUdR metabolism that includes IU-DNA and MMR. If the MMR parameters of this model are set to MMR deficient values, the model represents MMR^- ^malignant cells, otherwise, with wild type MMR parameters, the model represents MMR^+ ^normal cells. Such a model, or pair of models, could be used to develop novel multi-drug approaches to the treatment of MMR^- ^cancers. Specifically, the models could be used to predict how IUdR metabolism should be manipulated by drugs over time to maximize IU-DNA differences between MMR^- ^and MMR^+ ^cells at some time point. The timing of the maximum difference would then determine the best timing of acute irradiation to exploit the IU-DNA differences for a therapeutic gain. This treatment scenario is depicted in Figure 2.

**Figure 3:**
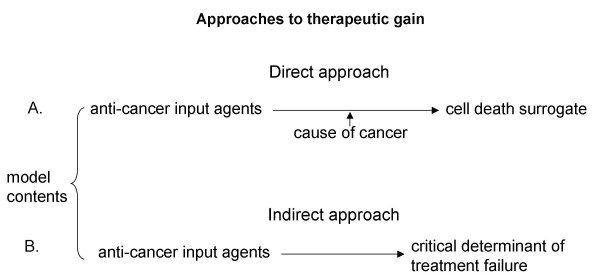
Model-based approaches to therapeutic gain. The direct approach (A) requires a model which includes the anti-cancer agents, the cause of the cancer, and an endpoint which correlates with the probability of cell death. The indirect approach (B) requires that the cancer be separable into poor and good prognosis groups and that the model includes the anti-cancer input agents and a critical determinant of treatment failure.

The problem statement outlined above is *direct *to the extent that its goal is selective killing of malignant versus normal cells. It requires a model that relates anti-cancer agents to an endpoint that is modulated by the cancer causing event and that correlates with cell death (Fig. 3A). These requirements define a class of cancer therapy problem statements with a common abstraction. The abstraction involves a pair of tissue specific dynamical system models of the same biological process, both manipulated synchronously through a set of common input functions. Subject to normal tissue toxicity constraints, the overall objective is to maximize the expectation of differential cell killing, defined as the probability of killing a malignant cell relative to the probability of killing a normal cell (i.e. the odds), or the log thereof. This defines a class of optimal control problems and a systems and control approach to therapeutic gain referred to hereafter as the *direct *approach.

To begin to formalize the direct approach mathematically, consider the pair of system models x˙
 MathType@MTEF@5@5@+=feaafiart1ev1aaatCvAUfKttLearuWrP9MDH5MBPbIqV92AaeXatLxBI9gBaebbnrfifHhDYfgasaacH8akY=wiFfYdH8Gipec8Eeeu0xXdbba9frFj0=OqFfea0dXdd9vqai=hGuQ8kuc9pgc9s8qqaq=dirpe0xb9q8qiLsFr0=vr0=vr0dc8meaabaqaciaacaGaaeqabaqabeGadaaakeaacuWG4baEgaGaaaaa@2E2E@^+ ^(*t*) = *f*(*x*^+^(*t*), *u*(*t*), *p*^+^, x0+
 MathType@MTEF@5@5@+=feaafiart1ev1aaatCvAUfKttLearuWrP9MDH5MBPbIqV92AaeXatLxBI9gBaebbnrfifHhDYfgasaacH8akY=wiFfYdH8Gipec8Eeeu0xXdbba9frFj0=OqFfea0dXdd9vqai=hGuQ8kuc9pgc9s8qqaq=dirpe0xb9q8qiLsFr0=vr0=vr0dc8meaabaqaciaacaGaaeqabaqabeGadaaakeaacqWG4baEdaqhaaWcbaGaeGimaadabaGaey4kaScaaaaa@3022@) and x˙
 MathType@MTEF@5@5@+=feaafiart1ev1aaatCvAUfKttLearuWrP9MDH5MBPbIqV92AaeXatLxBI9gBaebbnrfifHhDYfgasaacH8akY=wiFfYdH8Gipec8Eeeu0xXdbba9frFj0=OqFfea0dXdd9vqai=hGuQ8kuc9pgc9s8qqaq=dirpe0xb9q8qiLsFr0=vr0=vr0dc8meaabaqaciaacaGaaeqabaqabeGadaaakeaacuWG4baEgaGaaaaa@2E2E@^- ^(*t*) = *f*(*x*^-^(*t*), *u*(*t*), *p*^-^, x0−
 MathType@MTEF@5@5@+=feaafiart1ev1aaatCvAUfKttLearuWrP9MDH5MBPbIqV92AaeXatLxBI9gBaebbnrfifHhDYfgasaacH8akY=wiFfYdH8Gipec8Eeeu0xXdbba9frFj0=OqFfea0dXdd9vqai=hGuQ8kuc9pgc9s8qqaq=dirpe0xb9q8qiLsFr0=vr0=vr0dc8meaabaqaciaacaGaaeqabaqabeGadaaakeaacqWG4baEdaqhaaWcbaGaeGimaadabaGaeyOeI0caaaaa@302D@) for normal and malignant cells, respectively, where *x*(*t*) is a vector of state variables (e.g. metabolite and protein concentrations), *u*(*t*) is a vector of input time courses applied synchronously to both systems (e.g. extracellular drug concentrations), *p *is the set of model parameters that differ between the two cell types (parameters with identical values in both cell types are embedded in *f*), and the initial states of the normal and malignant cells are x0+
 MathType@MTEF@5@5@+=feaafiart1ev1aaatCvAUfKttLearuWrP9MDH5MBPbIqV92AaeXatLxBI9gBaebbnrfifHhDYfgasaacH8akY=wiFfYdH8Gipec8Eeeu0xXdbba9frFj0=OqFfea0dXdd9vqai=hGuQ8kuc9pgc9s8qqaq=dirpe0xb9q8qiLsFr0=vr0=vr0dc8meaabaqaciaacaGaaeqabaqabeGadaaakeaacqWG4baEdaqhaaWcbaGaeGimaadabaGaey4kaScaaaaa@3022@ and x0−
 MathType@MTEF@5@5@+=feaafiart1ev1aaatCvAUfKttLearuWrP9MDH5MBPbIqV92AaeXatLxBI9gBaebbnrfifHhDYfgasaacH8akY=wiFfYdH8Gipec8Eeeu0xXdbba9frFj0=OqFfea0dXdd9vqai=hGuQ8kuc9pgc9s8qqaq=dirpe0xb9q8qiLsFr0=vr0=vr0dc8meaabaqaciaacaGaaeqabaqabeGadaaakeaacqWG4baEdaqhaaWcbaGaeGimaadabaGaeyOeI0caaaaa@302D@, respectively. Here, normal cells may be dose-limiting marrow or gut cells, or they may be normal tissue counterparts of malignant cells. Since the two models are of the same biochemical system, they represent the same chemical species and thus have identical state space dimensions. Some of these dimensions will be more controllable than others, some will correlate with the probability of cell death more than others, and some, particularly those associated with major parameter differences, will separate values set initially to identical values in the two models more than others. For direct approach success, cancers of focus should be selected such that controllability, observability and separability are aligned within the same dimension(s) as much as possible.

### Indirect approach to therapeutic gain

Pro-B cell childhood acute lymphoblastic leukemia (ALL) [45] has event-free five year survival probabilities of 85–90% for the TEL-AML subtype and 20–40% for the BCR-ABL subtype [46]. Thus, TEL-AML1 ALL is essentially curable, BCR-ABL ALL is essentially non-curable, and both have pro-B cell origins. Suppose a biochemical system model existed that connected drug targets to a critical (see below) determinant of BCR-ABL treatment failure. Given such a model, a treatment failure risk state transfer strategy can be envisioned where the goal is to identify drug concentration time course schedules that transfer non-curable BCR-ABL initial states toward a target state region defined by the collection of curable TEL-AML1 states. In this scenario, if poor prognosis BCR-ABL patients could be brought to a state in the neighborhood of curable TEL-AML1 states, they might then be treated effectively from such a region by a standard TEL-AML1 therapeutic regimen.

**Figure 4:**
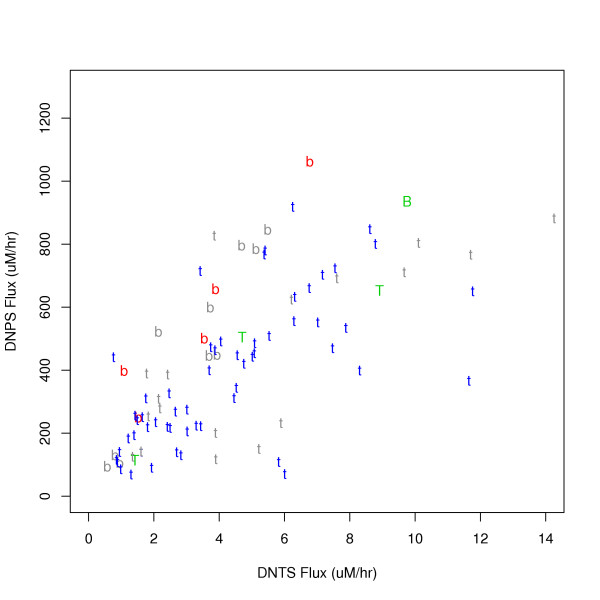
Folate model [3] DNPS vs. DNTS flux predictions based on the gene expression   data of Yeoh et al [36] (see Methods and [18]). Shown are TEL-AML1 patients   who had either a hematological relapse (HR) (green T), a continuous complete   remission (CCR) (blue t), or other outcome (gray t), and BCR-ABL patients   who had either a HR (red b), CCR (green B), or a censored, missing, or other   outcome (jointly, gray b).

Because effective combination TEL-AML1 therapies include methotrexate (MTX), it was hypothesized that differences in folate system states may exist between TEL-AML1 and BCR-ABL patients. To explore this hypothesis, the diagnostic bone marrow data of Yeoh et al [36] was used in conjunction with the folate model of Morrison and Allegra [3] to develop model-based steady state flux predictions of *de novo *purine synthesis (DNPS) versus *de novo *thymidylate synthesis (DNTS) as shown in Figure 4. In this plot, if the center of the TEL-AML1 patient states serves as the target of BCR-ABL patient state space trajectories, at first sight, it appears that BCR-ABL patients might benefit by replacements of MTX with ALIMTA, since ALIMTA inhibits glycinamide ribonucleotide formyltransferase and thus DNPS more so than DNTS [47]. This illustration of the concept of the indirect approach (Figure 3B) must, however, be carefully scrutinized before it is used to suggest new treatments. For example, if the one cured BCR-ABL patient (B in Fig. 4) serves as the guide, the other BCR-ABL patients should then be driven toward higher DNPS and higher DNTS (upper right in Fig. 4), rather than lower DNPS and higher DNTS (lower right in Fig. 4). Further, the TEL-AML1 patients with hematological relapses (T in Fig. 4) do not fall within the BCR-ABL cluster, and several TEL-AML1 patients who do fall within the BCR-ABL cluster were cured. Thus, closing differences in this plot may have no impact on treatment outcome. This suggests that the critical determinants of differences in treatment outcome lie downstream of the folate system, or are associated with other drugs in the TEL-AML1 regimen.

**Figure 5:**
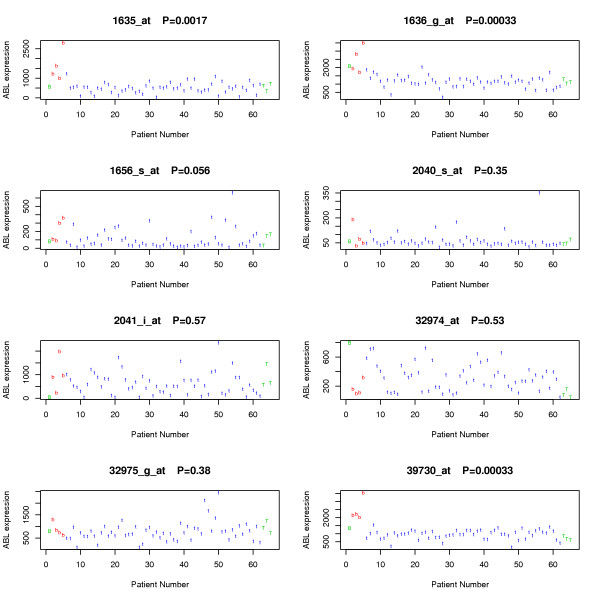
MAS5 U95av2 probeset summaries for ABL expression [36]. Symbols are as in Fig. 4. P values were obtained using the Rank Sum Test for differences between leukemias.

BCR-ABL is a prognostic determinant of treatment outcome because it defines the poor prognosis group. BCR-ABL is also likely to be the cause of BCR-ABL leukemias. However, this does not imply that successfully closing BCR-ABL activity differences cures patients. Above and beyond being prognostic, a *critical *determinant of treatment outcome can be defined as one which leads to improved treatment outcomes after difference closure. For example, because Gleevec does not cure BCR-ABL ALL, BCR-ABL is not a critical determinant of BCR-ABL ALL. However, since ABL expression (which is equivalent to BCR-ABL expression because probe sets are taken from the 3' end) is minimal for the cured BCR-ABL patient in 3 out of 4 probesets with P < .0.06 differences between leukemia subtypes, Fig. 5 does suggest (very mildly) that Gleevec combined with standard therapy could be beneficial, i.e. that BCR-ABL may be *conditionally *or *partially *critical. Thus, efforts to model successful drugs such as MTX in TEL-AML1 ALL might be productively complemented by parallel efforts to model candidate critical determinants of outcome.

The indirect approach is a generalization/abstraction of the BCR-ABL example given above. It can be characterized as an initial state/target state control problem, formulated within the framework of a dynamical system model. This is reminiscent of the problem of ballistic controllability that forms the foundation of the notions of controllability and reachability in modern systems theory. The ballistic control problem is one in which the objective is to find an input signal (i.e. control) that transfers a given initial state of the system to a specified final state in a given period of time. The basic idea of ballistic controllability is best understood for the simple system defined by the linear system model: dx(t)dt
 MathType@MTEF@5@5@+=feaafiart1ev1aaatCvAUfKttLearuWrP9MDH5MBPbIqV92AaeXatLxBI9gBaebbnrfifHhDYfgasaacH8akY=wiFfYdH8Gipec8Eeeu0xXdbba9frFj0=OqFfea0dXdd9vqai=hGuQ8kuc9pgc9s8qqaq=dirpe0xb9q8qiLsFr0=vr0=vr0dc8meaabaqaciaacaGaaeqabaqabeGadaaakeaadaWcaaqaaiabdsgaKjabdIha4naabmaabaGaemiDaqhacaGLOaGaayzkaaaabaGaemizaqMaemiDaqhaaaaa@3542@ = *B*(*t*)*u*(*t*) where x(t) is the state of the system (x ∈ R^n^), u(t) is the input (u ∈ R^m^, with m < n) and *B*(*t*) is a time varying system input matrix. The objective is to transfer the given initial state x(t_0_) = x_0 _to the specified final state x(t_f_) = x_f _over the time interval [t_0_, t_f_], i.e. the problem is to determine the *m *time courses in *u *such that x(tf)=∫t0tfB(τ)u(τ)dτ+x(t0)
MathType@MTEF@5@5@+=feaafiart1ev1aaatCvAUfKttLearuWrP9MDH5MBPbIqV92AaeXatLxBI9gBaebbnrfifHhDYfgasaacH8akY=wiFfYdH8Gipec8Eeeu0xXdbba9frFj0=OqFfea0dXdd9vqai=hGuQ8kuc9pgc9s8qqaq=dirpe0xb9q8qiLsFr0=vr0=vr0dc8meaabaqaciaacaGaaeqabaqabeGadaaakeaacqWG4baEdaqadaqaaiabdsha0naaBaaaleaacqWGMbGzaeqaaaGccaGLOaGaayzkaaGaeyypa0Zaa8qCaeaacqWGcbGqdaqadaqaaGGaciab=r8a0bGaayjkaiaawMcaaiabdwha1naabmaabaGae8hXdqhacaGLOaGaayzkaaGaemizaqMae8hXdqNaey4kaSIaemiEaG3aaeWaaeaacqWG0baDdaWgaaWcbaGaeGimaadabeaaaOGaayjkaiaawMcaaaWcbaGaemiDaq3aaSbaaWqaaiabicdaWaqabaaaleaacqWG0baDdaWgaaadbaGaemOzaygabeaaa0Gaey4kIipaaaa@4E4B@. For this problem, we introduce the *controllability gramian *W(t0,tf)=∫t0tfB(τ)B′(τ)dτ
MathType@MTEF@5@5@+=feaafiart1ev1aaatCvAUfKttLearuWrP9MDH5MBPbIqV92AaeXatLxBI9gBaebbnrfifHhDYfgasaacH8akY=wiFfYdH8Gipec8Eeeu0xXdbba9frFj0=OqFfea0dXdd9vqai=hGuQ8kuc9pgc9s8qqaq=dirpe0xb9q8qiLsFr0=vr0=vr0dc8meaabaqaciaacaGaaeqabaqabeGadaaakeaacqWGxbWvdaqadaqaaiabdsha0naaBaaaleaacqaIWaamaeqaaOGaeiilaWIaemiDaq3aaSbaaSqaaiabdAgaMbqabaaakiaawIcacaGLPaaacqGH9aqpdaWdXbqaaiabdkeacnaabmaabaacciGae8hXdqhacaGLOaGaayzkaaGafmOqaiKbauaadaqadaqaaiab=r8a0bGaayjkaiaawMcaaiabdsgaKjab=r8a0bWcbaGaemiDaq3aaSbaaWqaaiabicdaWaqabaaaleaacqWG0baDdaWgaaadbaGaemOzaygabeaaa0Gaey4kIipaaaa@4AAB@. If *W*(*t*_0_, *t*_*f*_) is full rank, then there exists an input signal vector *u*(*t*) that transfers x(t_0_) = x_0 _to x(t_f_) = x_f _. In fact, u(t) = B'(t)η where η = W(t_0_, t_f_)^-1^(x_f_-x_0_), i.e. the desired state transfer input signal is determined uniquely by the parameters of the problem. This simple problem can be easily extended to the class of linear systems: x˙
 MathType@MTEF@5@5@+=feaafiart1ev1aaatCvAUfKttLearuWrP9MDH5MBPbIqV92AaeXatLxBI9gBaebbnrfifHhDYfgasaacH8akY=wiFfYdH8Gipec8Eeeu0xXdbba9frFj0=OqFfea0dXdd9vqai=hGuQ8kuc9pgc9s8qqaq=dirpe0xb9q8qiLsFr0=vr0=vr0dc8meaabaqaciaacaGaaeqabaqabeGadaaakeaacuWG4baEgaGaaaaa@2E2E@(*t*) = *A*(*t*)*x*(*t*) + *B*(*t*)*u*(*t*)[27] and to the general class of nonlinear systems that are linear in the control effort *u*(*t*): x˙
 MathType@MTEF@5@5@+=feaafiart1ev1aaatCvAUfKttLearuWrP9MDH5MBPbIqV92AaeXatLxBI9gBaebbnrfifHhDYfgasaacH8akY=wiFfYdH8Gipec8Eeeu0xXdbba9frFj0=OqFfea0dXdd9vqai=hGuQ8kuc9pgc9s8qqaq=dirpe0xb9q8qiLsFr0=vr0=vr0dc8meaabaqaciaacaGaaeqabaqabeGadaaakeaacuWG4baEgaGaaaaa@2E2E@(*t*) = *f *(*x*(*t*)) + g(*x*(*t*))*u*(*t*) [28]. Important questions remain. For example, should the system state be clamped to lie within the target state region while applying standard therapy? Or should it be released to avoid interference between clamp control efforts and standard therapy control efforts? Or should a compromise between these two extremes be sought?

## Discussion

In the **direct **approach, the model includes the cause of cancer, and depending on the state of the modeled cancer cause, the model represents either a malignant or normal tissue cell. Here, the "cause of cancer" is the event that the therapy is being designed to exploit for a differential cell killing effect. Although secondary events essential to life-limiting malignant subpopulations are of interest, particularly with respect to drug resistance, the earlier the pivotal modeled event is in the cancer progression process the better, as the risk of cancer recurrence is greater if only a subpopulation of the malignancy is therapeutically eliminated.

The direct approach can also be applied to tumors with defective checkpoint function due to inactivation or deletion of the RB protein. After DNA damage, cells of these tumors progress into S phase and complete DNA replication without delays. If patients with such tumors are treated with an inhibitor of cdk2 to trigger a G1 checkpoint response, even in the absence of DNA damage, normal RB^+ ^cells will arrest at the G1 checkpoint and transformed RB^- ^cells will progress into S phase. Therefore, transformed cells become potentially susceptible to selective killing by S phase-selective cytotoxic agents. Thus, the cause of the cancer (in this instance a checkpoint defect) can be selectively exploited to cause differential cell killing. Although it is unlikely that the resulting selectivity of this approach will be complete, since, at the time of treatment with the second drug, some normal cells will be in S phase and will be killed while other tumor cells will be in other phases of the cell cycle and will escape, the relative timing of the two drugs could be optimized to maximize therapeutic gain. Mechanistically accurate models of the cell cycle [4] could facilitate such optimizations.

If the direct approach were applied to the treatment of BCR-ABL leukemias instead of the indirect approach, our goal would be to re-channel the anti-apoptotic BCR-ABL signal [48] into a pro-apoptotic signal, rather than merely block it as might be accomplished by a drug like Gleevec. Indeed, for the direct approach, one can argue that it is advantageous to exaggerate (rather than annihilate) differences between malignant and normal cells. For example, in the case of RB inactivated tumors, a larger S-phase fraction is expected in malignant versus normal cells due to some background G1 delays in normal cells, and this difference is exaggerated by a cdk2 inhibitor. Similarly, methoxyamine (MX) can be used to exaggerate DNA repair system competency differences between MMR^- ^malignant versus normal cells by further inhibiting base excision repair (BER) [49]. How BCR-ABL tyrosine kinase activity might be increased, and how it might be re-channeled to actively kill rather than protect cells, remain two unanswered and difficult questions. The direct approach was useful to us here to the extent that it led us to realize that these two questions are important.

The main difficulty with the **indirect **approach is that the critical determinants of treatment failure must be known and included in the model. If they are not (because the scope of the model is not extensive enough), treatments may end up targeting closures of non-critical state space differences. To find critical state space dimensions, a reasonable strategy is to start with models of the targets of drugs which cure patients in the good prognosis group and move modeling efforts inwards toward apoptosis, since somewhere along this path, as seen in patients, critical differences are likely to exist. Thus, for the BCR-ABL/TEL-AML1-folate model example given above, since the MTX target system is already in the model, the next logical modeling extensions might be to include DNA damage repair and the activation of apoptosis. Alternatively, or in parallel to such efforts, models of the target systems of other drugs present in the standard TEL-AML1 therapeutic regimens should also be developed.

At one extreme, attempts to use TEL-AML1 patients as guides for say lung cancer patients would likely fail, either because the dimensionality of the difference space is too high to find the critical subspaces, or because the dimensionality of the critical subspace is too high to make the needed difference closures. At the opposite extreme, hypothetically, there could be a single dimension difference space, and thus too, a single critical dimension. The closer we can come to this ideal, and the greater the differences in treatment outcomes, the greater the likelihood of indirect approach success. In addition to the BCR-ABL/TEL-AML example, diffuse large B-cell lymphoma patients are a candidate for this approach since they can be fractionated into good and poor prognosis groups using DNA microarray data [50-52] or 6 genes [53]. The idea of using DNA microarrays both to fractionate patients into poor and good prognosis groups, and to define the poor prognosis patient initial and target states, is potentially quite powerful. Additional indirect approach research therefore seems warranted.

In an abstract sense, normal tissues can be viewed as cancers that are cured without therapy 100% of the time, i.e. as solved cancer counterparts for non-curable cancers of the same tissue origin. Using such guides in the indirect approach, the goal is to make cancer cells like normal cells, namely, cured but not killed; e.g. Gleevec usage is an instance of this form of the indirect approach, and it appears that Gleevec does not kill BCR-ABL leukemic stem cells [54]. When using normal tissue counterpart guides in the indirect approach, an infinite clamp is called for once the target state region has been reached, since there are no risks of treatment interactions given that the standard regimen is null in this case. Thus, for the indirect approach, normal tissue guides yield lifelong cancer treatment solutions. In contrast, since cured cancer guides are killed by standard therapies, the cancer cells of the non-curable patients should follow suit, i.e. using cured cancer guides in the indirect approach should yield finite treatment solutions. This is an expected advantage of using *bona fide *solved cancer counterparts rather than normal tissues as indirect approach guides.

A logical research sequence in systems cancer biology is to: a) select a relatively well-understood cancer and a treatment strategy (preferably the direct approach); b) build an initial model of processes that are most pertinent to the selected cancer; c) iteratively improve the model through experimental validations and model expansions; and d) develop model-based optimal control solutions to the corresponding optimal control problems. Currently, we are focusing on steps a) and b) for MMR^- ^cancers, though we are also interested in BCR-ABL leukemias.

## Conclusion

Because the scope of a model is determined by its intended uses, models should be developed with their ultimate uses in mind. As biological knowledge, the number of anti-cancer agents, and the number of possible measurements, continues to grow, mathematical models of cancer relevant processes will find uses in the design of state feedback-based clinical trials. Such model-based, systems and control oriented therapies will be individualized via polymorphism-based model parameter perturbations and patient differences in initial states (see Figure 1). Conceptual frameworks such as those presented here are needed to accomplish these goals.

## Competing interests

The author(s) declare that they have no competing interests.

## Authors' contributions

TR performed the computations and wrote the first two drafts, KAL wrote sections requiring control system expertise, RCJ contributed the cell cycle inhibition example in the Discussion, and WDS improved the writing in all of the sections.

## Pre-publication history

The pre-publication history for this paper can be accessed here:
